# Risk factors of anemia among pregnant women attending antenatal care in health facilities of Eastern Zone of Tigray, Ethiopia, case-control study, 2017/18

**DOI:** 10.11604/pamj.2019.34.121.15999

**Published:** 2019-10-31

**Authors:** Kidanemaryam Berhe, Berhane Fseha, Gebrehiwot Gebremariam, Hirut Teame, Natnael Etsay, Guesh Welu, Tesfay Tsegay

**Affiliations:** 1 Department of Nutrition and Dietetics, School of Public Health, Mekelle University, Mekelle, Ethiopia; 2 Department of Public Health, Adigrat University, Adigrat, Ethiopia; 3 Department of Midwifery, Adigrat University, Adigrat, Ethiopia; 4 Department of Midwifery, Aksum University, Axum, Ethiopia; 5 Department of Nursing, Adigrat University, Adigrat, Ethiopia

**Keywords:** Anemia, risk factors, pregnant women, Tigray

## Abstract

**Introduction:**

Worldwide the average prevalence of anemia among pregnant women is 38.2% and in Ethiopia, the average prevalence of anemia among pregnant women is 22%. The aim of this research was to identify risk factors of anemia among pregnant women in Eastern Zone of Tigray, Ethiopia.

**Methods:**

A case-control study was conducted among 600 (150 cases and 450 controls) pregnant women in 2017/18. Pregnant women with a hemoglobin level below 11 g/dl were cases (anemic) and those with hemoglobin >11 g/dl were controls (non-anemic). Data were collected using structured questionnaire and SPSS version 20 was used for analysis. Bivariate and multivariate logistic regression model was used to identify the risk factors for anemia among pregnant women. P-value <0.05 and adjusted odds ratio with a 95% confidence interval were used to assess the association.

**Results:**

Intestinal parasites (adjusted odds ratio (AOR)=3.4; 95% confidence interval (CI): 1.2, 17.9), farmer occupation (AOR=3, 95% CI: 1.4, 10.8), unprotected sources of drinking water (AOR=3; 95% CI: 1.7, 16.9), drinking coffee/tea with or immediately after meal daily (AOR=1.9; 95%CI: 1.04, 8.7) and diet diversity score (DDS) of less than 3 (AOR=3; 95% CI: 1.5, 5.5) were statistically significant for anemia among pregnant women.

**Conclusion:**

In this study, the risk factors for anemia among pregnant women were intestinal parasites, mother farmer occupation, unprotected source of drinking water, drinking coffee or tea with a meal or immediately after meal and low diet diversification score. Therefore, nutritional intervention should consider the above-identified risk factors.

## Introduction

World health organization (WHO) defines anemia in pregnancy as a reduction in the oxygen-carrying capacity of the blood as a result of fewer circulating erythrocytes than normal or a decrease in the concentration of hemoglobin level below 11 g/dl. For the production of hemoglobin and red blood cells, our body needs different micronutrients like iron, vitamin B12, folate and others from the food we eat. If there is a low intake of Iron (<20mg/day), low folate intake (<70mg/day), low vitamin B12 concentration (<150pg/ml) then there could be different types of anemia like microcytic and megaloblastic anemia [1]. According to the World Health Organization’s estimation, anemia is considered to be a mild public health problem if the prevalence of anemia is between 5% and 19.9%, a moderate public health problem if the prevalence is between 20% and 39.9% and a severe public health problem if the prevalence is 40% and above [2]. Worldwide, about 32.4 million (38.2 %) pregnant women are affected by anemia and the burden is high in developing countries especially in South East Asia and Africa [3]. In Ethiopia, according to Ethiopia demographic and health survey 2011 (EDHS 2011), the average prevalence of anemia among pregnant women was 22% and there was a difference from region to region which may result from a number of causes' variations from locality to locality [4].

Anemia affects human health, social and economic development which results in a loss of billions of dollars annually [5]. Anemia consequences on maternal health include less exercise tolerability, puerperal infection, thromboembolic problems, postpartum hemorrhage, pregnancy-induced hypertension, placenta previa and cardiac failure [6-8]. Anemia can contribute up to 40% of maternal mortality in which the majority of this happens in underdeveloped countries [9]. World Bank development report 2014 showed that about 420 Ethiopian mothers die per 100,000 live births every year. Likewise, anemia is a risk factor for intrauterine growth retardation and subsequent low birth weight, preterm delivery, prenatal death, poor cognitive and motor development [10,11]. Anemia among pregnant women is still a public health problem in Ethiopia. Moreover, there was limited evidence on the risk factors of anemia among pregnant women in the study area. Therefore, this study was conducted to fill this gap by identifying the risk factors of anemia among pregnant women. The finding of this study is important for evidence-based intervention and for the achievement of the 2025 goal, which is a 50% reduction in anemia [12].

## Methods

**Study area:** the study was conducted in the Eastern Zone of Tigray Region, Ethiopia which is located at 870km from the capital city of Ethiopia (Addis Ababa). In 2017/2018, according to the zone health office, the total population of the zone was 774,155. About 379,336 (49%) were males and the rest were females. There are two urban wereda (Districts) and seven rural wereda (Districts). In the study area, there are two general hospitals, five primary hospitals and 39 health centers (4 in urban and 35 in rural). The major agricultural products found in Eastern Zone of Tigray region are cereals, grains, vegetables, fruits, roots, honey, animal products like meat, poultry, milk and milk products [13].

**Study design:** a facility-based unmatched case-control study design was applied.

**Study period:** the study period was from January 2017 to January 2018.

**Source population:** all pregnant women attending antenatal care (ANC) in health facilities of Eastern Tigray Region.

**Study population:** all pregnant women attending ANC in selected health facilities during the data collection period.

**Eligibility criteria:** all pregnant women who attend their antenatal care in health facilities during their first visit and those who are residents for a minimum of six months in the study area were included. Pregnant women who are severely ill were excluded due to the difficulty to get full information from severe ill pregnant women and it is again unethical to collect from severely ill pregnant women. Pregnant women in the second and above ANC visits were excluded because these pregnant women received iron folate supplementation from the previous ANC visit/s which can increase their hemoglobin level and makes difficult to know their anemia status and risk factors before the iron folate supplementation.

**Sample size determination:** the sample size was calculated by using Epi-Info version 7.1.5.2. To determine the sample size intestinal parasite was taken as a risk factor for anemia among Ethiopia pregnant women and this variable (intestinal parasite) was selected among other significant variables because it gave a large sample size. Expected frequency of intestinal parasite was 23.2% in anemic pregnant women and 8.9% in non-anemic pregnant women [14]. The following assumptions were considered; 95% confidence interval, 80% power and case to control ratio 1:3. Considering 5% non-response rate and a design effect of 2, the sample size was 600; of these 150 were cases and 450 were controls.

**Sampling technique:** a sample was taken from both urban and rural health facilities. The simple random method was used to select districts/wereda and health facilities from the selected districts/wereda. Out of 46 health facilities, a total of 16 health facilities were randomly selected. Total numbers of study participants from each health facility were taken based on their proportion to population size (PPS). Participants were recruited at the antenatal care unit using consecutive sampling technique until the required sample size was attained.

**Standard definition:** anemia in pregnant women: hemoglobin level of < 11 g/dl [15]. Cases: pregnant women with a hemoglobin level of < 11 g/dl. Controls: pregnant women with a hemoglobin level of > 11 g/dl.

**Data collection tools and procedures:** data were collected using a structured questionnaire. Hemoglobin status was determined and those with the hemoglobin level below 11 g/dl were selected as cases (anemic) and those with hemoglobin level > 11 g/dl were selected as controls (non-anemic) [15]. The questionnaire includes socio-demographic and economic, health and diseases, dietary habit and nutrition, hygiene and sanitation-related factors. Stool sample (5 gm) was collected from each study participant using clean, wide-mouthed and leak-proof stool cup. Then, a stool sample was examined for parasites. The dietary score was assessed by using a single 24-hour dietary recall. All the foods and the liquids consumed a day before the study were categorized into 9 food groups. Consuming a food item from the group was assigned a score of ''1'' and if no food was consumed a score of ''0'' was given. Accordingly, a DDS out of 9 points was computed by combining the values of all the groups.

**Data quality assurance:** first, the questionnaire was prepared in English and then translated to the local language Tigrigna and back to English to check for consistency by language experts. A pre-test was conducted in 5% of the sample size before the actual data collection. The training was given for data collectors and supervisors. During the data collection, data collectors were strictly supervised. A principle of standard operation procedure (SOP) was applied in the laboratory test and reagents for manufacturing, expiry date, and proper storage was checked to ensure quality. The sample was processed immediately after collecting from the study participants. Supervisors checked out the completeness of filled questionnaires. Any error, ambiguity, incompleteness, or other encountered problems was addressed. The overall data collection process was controlled by a principal investigator and co-investigators.

**Data analysis:** data were entered into Epi Data version 3.1 for cleaning and were exported to SPSS version 20 for analysis. The outcome variable was dichotomized into 1=case and 0=control. Descriptive statistics were computed for the study variables. Bivariate logistic regression analysis was performed for independent variables with the outcome of interest and p-value < 0.25 was used as a cut off point for statistically significance. Finally, multivariable logistic regression analysis was done to identify the risk factors of anemia among pregnant women. In multivariable logistic regression analysis variables with p-value < 0.05 was taken as statistically significant and adjusted odds ratio with its 95% CI was also reported. Multicollinearity was done using variance inflation factor (VIF) and there was no collinearity between the independent variables. Model goodness of fit was assessed using the Hosmer-Lemeshow test and the p-value of the model fitness test was 0.127.

**Ethical consideration:** this study was reviewed by the Research Ethics Committee, College of Medicine and Health Science and Office of Research and Community Services in Adigrat University and ethical approval was obtained from this institution. The reference number of this study was AGU/CMHS/030/09. Written permission was obtained from the Tigray Regional Health Bureau, district/wereda health offices and selected health facilities. Further, study participants were briefed about the main objective of the study. Participants were informed that they have the full right to refuse to participate in the study or can interrupt/withdraw if they want. Confidentiality of the information was assured and the privacy of the study participants was respected and kept as well. Informed consent was obtained from each study participant. At the last, pregnant women with anemia were treated based on the degree of severity of anemia and all pregnant women were counseled on personal hygiene, sanitation, nutritional practices and healthy lifestyle.

## Results

**Socio-demographic and economic-related factors:** a total of 600 pregnant women were included in this study (150 anemic and 450 non-anemic). Majority of the cases and controls were married and Tigrean in ethnicity. Pregnant women were 132(88%) in cases and 387(86%) pregnant women in controls were orthodox Christian. More than one-third of the cases and controls were a housewife in their occupation. Half of the cases and a quarter of the controls were not educated, 57(38%) of husbands of the cases and 146(32.5%) of husbands of the controls were not educated. About 42.7% of the cases and 50.7% of the controls were in the age of 20-30 years. About 87(58%) of the cases were from rural areas and 260(57.8%) of the controls were from urban areas. Monthly family income was <500 Ethiopian birr in 65(43.3%) of the cases and 140(31.1%) of the controls. About 20(13.3%) of cases and 147(32.7%) of controls had monthly income of more than 1500 Ethiopian birr.

**Hygiene and sanitation-related factors:** around one-fifth of the cases and one-third of the controls didn't have a latrine. Eighty-one(54%) of the cases and 55(12.2%) wash their hand using only water. About 20.7% of the cases and 2.9% of the controls use unprotected well/spring/river/stream as a source of water for drinking. About 52(34.7%) of cases and 201(44.1%) of controls disposed of wastes by a municipality.

**Health status and lifestyle-related factors:** in 51(30%) of the cases and 36(8%) of the controls, intestinal parasites were identified using stool examinations. Maternal mid-arm circumference (MUAC) was < 19 cm in 20.7% of the cases and 2.7% of the controls. About 116(77.3%) of the cases and 111(24.7%) of the controls were fasting during their pregnancy and the reason for fasting was religion purpose for all fasting pregnant women (Table 1). WHO diet diversity score of the pregnant women was assessed using 24-hours recall method and nine food groups were considered for assessing the diet diversity score. Accordingly, WHO diet diversity score was < 3 score in 65.3% of the cases and 10.9% of the controls respectively.

**Table 1:** Health status and lifestyle of the pregnant women in Eastern Zone of Tigray, Ethiopia, 2017/18

S.no	Variable	Anemic pregnant women (n=150)	Non-anemic pregnant women (n=450)
1	**Number of meals per day (usual)**		
	2 meals	63(42%)	39(8.7%)
	3 meals	43(28.7%)	169(37.6%)
	4 meals	19(12.7%)	173(38.4%)
	>=5 meals	25(16.7%)	69(15.3%)
2	**Drinking tea/coffee immediately or within meals**		
	No	31 (20.7%)	350 (77.8%)
	Yes	119 (79.3%)	100 (22.2%)
3	**Number of abortion**		
	No abortion	127(84.7%)	415(92.2%)
	1 times	13(8.7%)	24(5.3%)
	2 times	10(6.6%)	11(2.4%)
4	**Presence of diseases**		
	No	129(86%)	419(93.1%)
	Yes	21(14%)	31(6.9%)
5	**Frequency of dairy products consumption**		
	Never took	25(16.7%)	9(2%)
	Every other day	53(35.3%)	16(3.6%)
	One per week	21(14%)	144(32%)
	One per two weeks or above	51(34%)	281(62.4%)
6	**Frequency of organ meat consumption**		
	One per week	30(20%)	151(33.6%)
	One per two weeks or above	120(80%)	299(66.4%)

**Risk factors for anemia during pregnancy:** to identify the risk factors of anemia during pregnancy both bivariate and multivariate logistic regression were applied. Factors with a p-value of less than 0.25 in bivariate analysis (Table 2) were entered simultaneously to multivariate logistic regression analysis. To avoid the risk of overfitting in the multivariate model, the most significant variables in bivariate analysis were selected. In multivariate analysis, only five factors with a p-value of less than 0.05 were remained to be independently significantly associated factors for anemia during pregnancy. Interaction test was done and there was no interaction among the variables. According to this study, intestinal parasites were significantly higher in cases (anemic pregnant women) as compared to controls (adjusted odds ratio (AOR) =3.4; 95% confidence interval (CI): 1.2, 17.9). Pregnant women with farmer occupation were higher in cases than controls (AOR=3, 95% CI: 1.4, 10.8). Unprotected sources of drinking water were higher in cases as compared to controls (AOR=3; 95% CI: 1.7, 16.9). Coffee/tea-drinking with/immediately after meals daily was found as a risk factor for anemia during pregnancy (AOR=1.9; 95% CI: 1.04, 8.7). It was also found that diet diversity score (DDS) of less than 3 was statistically significant for anemia during pregnancy (AOR=3; 95%CI: 1.5, 5.5) (Table 3).

**Table 2:** Bivariate logistic regression analysis result of variables (p-value<0.25) for anemia in pregnant women in Eastern Zone of Tigray, Ethiopia, 2017/18

S.no	Variable	Anemic pregnant women	Non-anemic pregnant women	COR	95% CI of COR	
					Lower	Upper
1	**Presence of intestinal parasites**					
	No (ref.)	99	414	1		
	Yes	51	36	5.9	3.7	9.3
	Total	150	450			
2	**MUAC**					
	<19cm	31	12	14	7.1	29.5
	19-23cm	54	75	4	2.6	6.2
	>23cm (ref.)	65	363	1		
	Total	150	450			
3	**Family size**					
	<=4 (ref.)	73	378	1		
	>4	77	72	5.5	3.7	8.3
	Total	150	450			
4	**Educational status of the pregnant woman**					
	No education	76	120	2.5	1.5	4.4
	Primary school	28	142	0.8	0.4	1.5
	Secondary school	24	99	0.9	0.5	1.9
	Above secondary school (ref.)	22	89	1		
	Total	150	450			
5	**Family income**					
	<=500birr	65	140	3.4	1.9	5.9
	501-1000birr	50	111	3.3	1.8	5.9
	1001-1500birr	15	52	2.1	1.01	4.5
	>1500birr (ref.)	20	147			
	Total	150	450			
6	**Source of drinking water**					
	Dwelling piped supply (ref.)	22	139	1		
	Tap/public	38	96	2.5	1.4	4.5
	Borehole	39	152	1.6	0.9	2.9
	Protected well/spring	20	50	2.5	1.3	5
	Unprotected well/spring, river, stream lake or dam	31	13	15	6.9	33.2
	Total	150	450			
7	**Coffee/tea-drinking with/immediately after meals daily**					
	Yes	119	100	13.4	8.5	21.1
	No (ref.)	31	350	1		
	Total	150	450			
8	**Presence of disease**					
	Yes	21	31	2.2	1.22	3.9
	No (ref.)	129	419	1		
	Total	150				
9	**WHO DDS**					
	<=3 (low)	98	49	28	10.8	62.2
	4-5 (medium)	37	111	6.4	3.4	12.2
	>=6 (recommended) (ref.)	15	290	1		
	Total	150	450			

**Table 3:** Multivariate logistic regression analysis result of variables for anemia in pregnant women in Eastern Zone of Tigray, Ethiopia, 2017/18

S.no.	Variable	Anemic	Non-anemic	AOR	95% CI of AOR	
					Lower	Upper
1	**Presence of intestinal parasites**					
	No (ref.)	99	414	1		
	Yes	51	36	3.4	1.2	17.9**
	Total	150	450			
2	**Occupation of the pregnant woman**					
	Civil servant (ref.)	23	152	1		
	Trade	33	51	1.5	0.84	4.9
	Household	52	194	1.5	0.54	6.4
	Farmer	42	53	3	1.4	10.8*
	Total	150	450			
3	Source of drinking water*					
	Dwelling piped supply (ref.)	22	139	1		
	Tap/public	38	96	0.13	0.3	1.2
	Borehole	39	152	0.3	0.1	1.3
	Protected well/spring	20	50	1.2	0.23	6.2
	Unprotected well/spring, river, stream lake or dam	31	13	3	1.7	16.9*
	Total	150	450			
4	**Coffee/tea-drinking with/immediately after meals daily**					
	Yes	119	100	1.9	1.04	8.7**
	No (ref.)	31	350	1		
	Total	150	450			
5	**WHO DDS**					
	<=3 (low)	98	49	3	1.5	5.5**
	4-5 (medium)	37	111	2	1.03	9.8
	>=6 (recommended) (ref.)	15	290	1		
	Total	150	450			

*=p-value<0.05, **=p-value<0.001

## Discussion

Anemia is a public health problem throughout the world, particularly in developing countries including Ethiopia which can affect human health, social and economic development. The aim of this study was to identify the risk factors for anemia during pregnancy in the Eastern Zone of Tigray, Ethiopia by considering as many factors as possible. For this purpose, data were collected from pregnant women who visited health facilities for antenatal care services in the Eastern Zone of Tigray. This study identified that intestinal parasites, mother occupation (farmer), an unprotected water source for drinking, drinking of coffee/tea immediately/with meals and low diet diversification score (DDS) were the main risk factors for anemia during pregnancy in Eastern Zone of Tigray. The five identified factors in this study are discussed as follows. In this study, a statistically significant association was found between intestinal parasites (Hookworm, *Ascaris lumbricoides*, *Trichuris trichiuria*) and anemia in pregnancy. This is consistent with studies conducted in Vietnam [16], Nigeria [17], Benin [18], Ethiopia [19], Gilgel Gibe Dam area [20], Southeast Ethiopia [21], Southern Ethiopia [22], Northwest Ethiopia [23], Wolayta Sodo town [24]. Again, it was estimated that between a quarter and a third of pregnant women in sub-Saharan Africa are infected with intestinal parasites and at risk of preventable intestinal parasites-related anemia. Intestinal parasites cause blood loss, putting the women and the fetus at high risk for anemia. The worm in the intestine may cause intestinal necrosis and blood loss as a result of the attachment to the intestinal mucosa and lead to anemia [25,26].

This study showed that mother occupation (being farmer) was significantly associated with anemia in pregnancy. About 42(28%) of pregnant women from cases and 53(11.8%) pregnant women from controls were a farmer in occupation. This could be related to lack of information about adequate and appropriate nutritional practices during pregnancy, economic factors, inaccessibility and less utilization of health care services [4,27]. A water source for drinking (unprotected spring/stream/river/lake) was significantly associated with anemia during pregnancy. In this study 31(20.7%) of the cases and 13(2.9%) of the controls were drinking water from unprotected spring/stream/river/lake. This may lead to different infections which in turn cause depletion of the iron and other micronutrients and development of anemia. The other variable that showed significant association with anemia during pregnancy in this study was drinking of coffee/tea with a meal or immediately after a meal. Similar findings were reported from studies done in Pakistan [28] and Oromia region [29]. Anemia can be affected by the presence of anti-nutritional factors like tannin and caffeine found in tea and coffee respectively [30]. Tea and coffee tend to decrease iron absorption by 60% and 50% respectively [8,31,32]. The effect of tea on the absorption of non-hem iron was ascribed to the formation of insoluble iron tannate complexes [29]. Moreover, this study showed that a low diet diversity score was a significant factor for anemia during pregnancy. This finding was in agreement with findings of studies done in Vietnam [16], underdeveloped area [33], Tigray region [34], Mekelle town [35] and southern Ethiopia [22]. Low dietary diversity leads to a deficiency of minerals and vitamins that may affect iron status. This might be due to the fact that pregnancy is a special period with increased energy and nutrient requirements, which can be fulfilled with increased meal frequency [36]. Consequently, pregnant women are advised to eat more diversified diet than usual [35]. Many studies in the different area showed as factors like women educational status [37], MUAC [6] and lack of toilet [38] were significant factors for anemia during pregnancy but in this study, these factors are not statistically significant. This could be due to the socio-economic changes and the difference in the study area.

## Conclusion

In this study, the risk factors for anemia among pregnant women were identified. The risk factors were intestinal parasites, mother occupation (being farmer), unprotected source of drinking water, drinking coffee or tea with a meal or immediately after meal and low diet diversification score. The possible recommendations to address these identified risk factors include: periodic deworming and community education on sanitary practices to control and prevent the intestinal parasites; promotion of diversified diet; promotion of safe water, avoiding of drinking coffee or tea with meal or immediately after meal but they can drink coffee or tea after two hours of meal or two hours prior to meal and emphasis should be given for rural areas.

### What is known about this topic

- Prevalence of anemia among pregnant women is known at the national level;
- Prevalence of anemia among pregnant women is known at the regional level but the risk factors for anemia in pregnant women were not known;
- The main strategy to prevent and control anemia among pregnant women is through the provision of iron folate supplementation during antenatal follow up.

### What this study adds

- Anemia among pregnant women was high among those who had intestinal parasites and those who used an unprotected source of water for drinking;
- Pregnant women who consumed low diversified diet were at high risk of anemia;
- Drinking coffee or tea with a meal or immediately after a meal increases the development of anemia during pregnancy.
